# Myxofibrosarcoma of the mandible: a case report and review of the literature

**DOI:** 10.1186/s12903-020-01094-7

**Published:** 2020-04-16

**Authors:** Zhengqiang Li, Xianwen Liu, Quanyin Zhang, Jie Zhang, Mingyi Huang, Shuguang Liu

**Affiliations:** Department of Oral and Maxillofacial Surgery, Stomatological Hospital of Southern Medical University, 366 south of Jiangnan Road, Guangzhou, 510280 China

**Keywords:** Head and neck, Mandible, Maxilla, Soft tissue tumor, Myxofibrosarcoma

## Abstract

**Background:**

Myxofibrosarcoma (MFS) is a soft tissue sarcoma that commonly occurs in late adult life. It is mainly located in the subcutaneous soft tissues of extremities characterized by a high recurrence rate at the original site. MFS of the head and neck is rare, while it occurs in the maxilla and mandible is extremely rare.

**Case presentation:**

We report a case of MFS of the mandible in a 51-year-old female who presented with a painless gingival swelling and mobile, super-erupted right mandibular second and third molars. Panoramic x-ray and maxillofacial CT revealed an ill-defined radiolucent lesion surrounding the mandibular molars giving a teeth-floating-in-air appearance. Histopathological examination showed scattered spindle and stellate cells with mild atypia distributed in the myxoid stroma. Only a few mitotic figures were identified and no area of tissue necrosis was found. The characteristic thin-walled and curvilinear vasculature were prominent. Immunohistochemistry analysis revealed the tumor cells being positive for vimentin and vascular CD31. CK, S-100, P63, HHF-35 stains were negative. The labeling index of Ki-67 was about 30%. Based on the histopathological and immunohistochemical examinations, the diagnosis of a low-grade MFS was established. This patient underwent a radical segmental excision with a 2-cm margin, supraomohyoid neck dissection and immediate reconstruction of the mandibular continuity defect with a fibular osteocutaneous free flap. This patient has been followed for 20 months to date and has remained disease free.

**Conclusions:**

This report describes a rare case of MFS of the mandible. Recognizing the histopathological features of MFS and applying the appropriate immunohistochemical examinations are crucial in establishing the correct diagnosis. Our case may provide diagnosis and treatment experiences of MFS occurs in the mandible.

## Background

Myxofibrosarcoma (MFS) is a fibroblast-derived sarcoma, which accounts for approximately 5–10% of all soft tissue malignant tumors [1]. The World Health Organization (WHO) defines MFS as the malignant fibroblastic neoplasm characterized by cellular pleomorphism, variably prominent myxoid stroma, and prominent elongated, thin-walled stromal blood vessels [2]. The mean age in patients with MFS is between the fifth and seventh decades [3]. Although some studies show a slight male predominance [4], the current evidence suggests no significant gender predilection [5]. About 77% of MFS cases occur in the extremities with a predilection for the upper extremities. Other areas of the body including the trunk (12%), retroperitoneum or mediastinum (8%) [4, 6], abdominal wall [7], heart [8] have also been reported. As a soft tissue tumor that mainly occurs in the subcutaneous tissue, MFS of the head and neck is quite rare. This case report describes the clinical features, histopathological and immunohistochemical examinations and treatment experience in managing this rare sarcoma.

## Case presentation

**Fig. 1:**
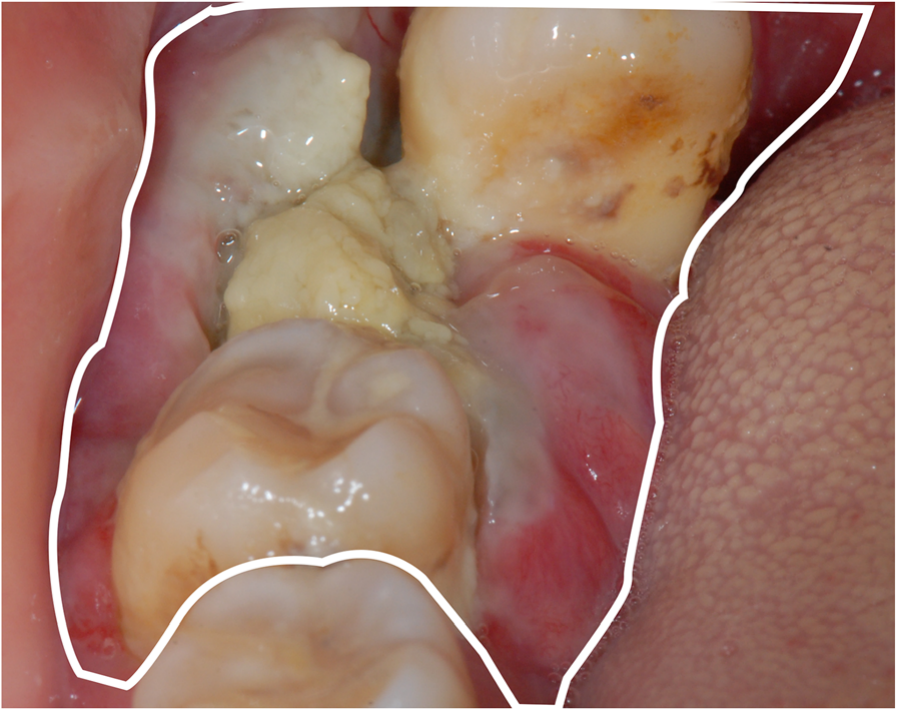
preoperative examinations. The figure showing apparent swelling of the gingiva lesion (the white box)

**Fig. 2:**
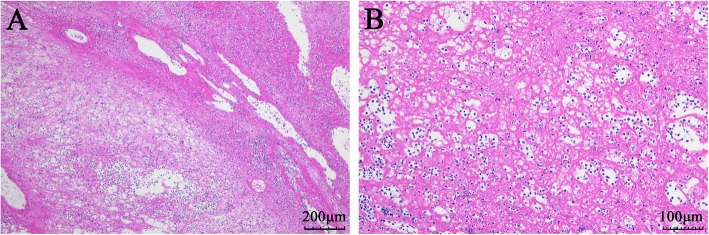
Hematoxylin-Eosin Staining of the gingiva lesion in pre-admission. The two images of **a** (×100) and **b** (×200) were suspicious for a diagnose of granulomatous lesion

This is a 51-year-old female who presented with a three-month history of an enlarging gingival mass of the right mandible. Head and neck evaluation revealed no facial asymmetry, cervical lymphadenopathy nor trismus. She denied pain, sensory alterations or bleeding associated with the lesion. The right mandibular second and third molars were super-erupted, grossly mobile, but not painful to palpation. The exophytic gingival lesion was covered with a yellowish pseudomembrane (Fig. 1). A slight cortical expansion was noted at the buccal vestibule. An outside dental provider performed an incisional biopsy of the gingival lesion prior to referring her to our institution for a higher level of care. The incisional biopsy performed suggested pyogenic granuloma as the diagnosis (Fig. 2a, b).

**Fig. 3:**
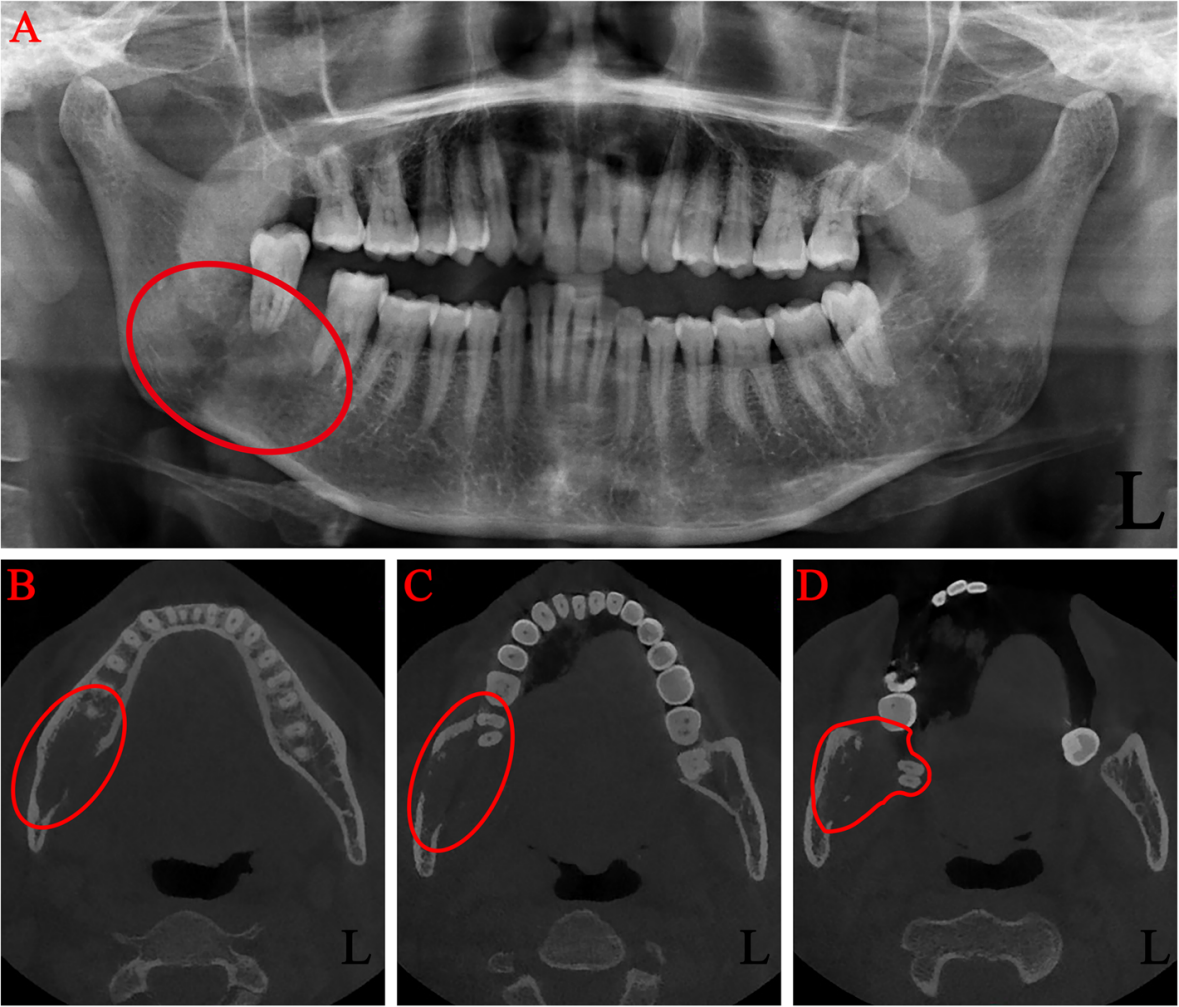
Preoperative images of radiological examinations. Panoramic radiograph (**a**) and computed tomography (**b**, **c**, **d**) showing a large area of right mandibular destruction (the red box)

On admission, imaging studies including panoramic radiograph and maxillofacial computed tomography (CT) without contrast were obtained. An ill-defined radiolucent lesion involving the right mandibular angle was noted. The lesion involved the entire bucco-lingual width of the right mandibular angle with extension through the lingual cortex. The right mandibular second and third molars were both super-erupted with a teeth-floating-in-air appearance (Fig. 3a, b, c, d). The outlines of the mandibular canal were obscured by the intraosseous lesion.

**Fig. 4:**
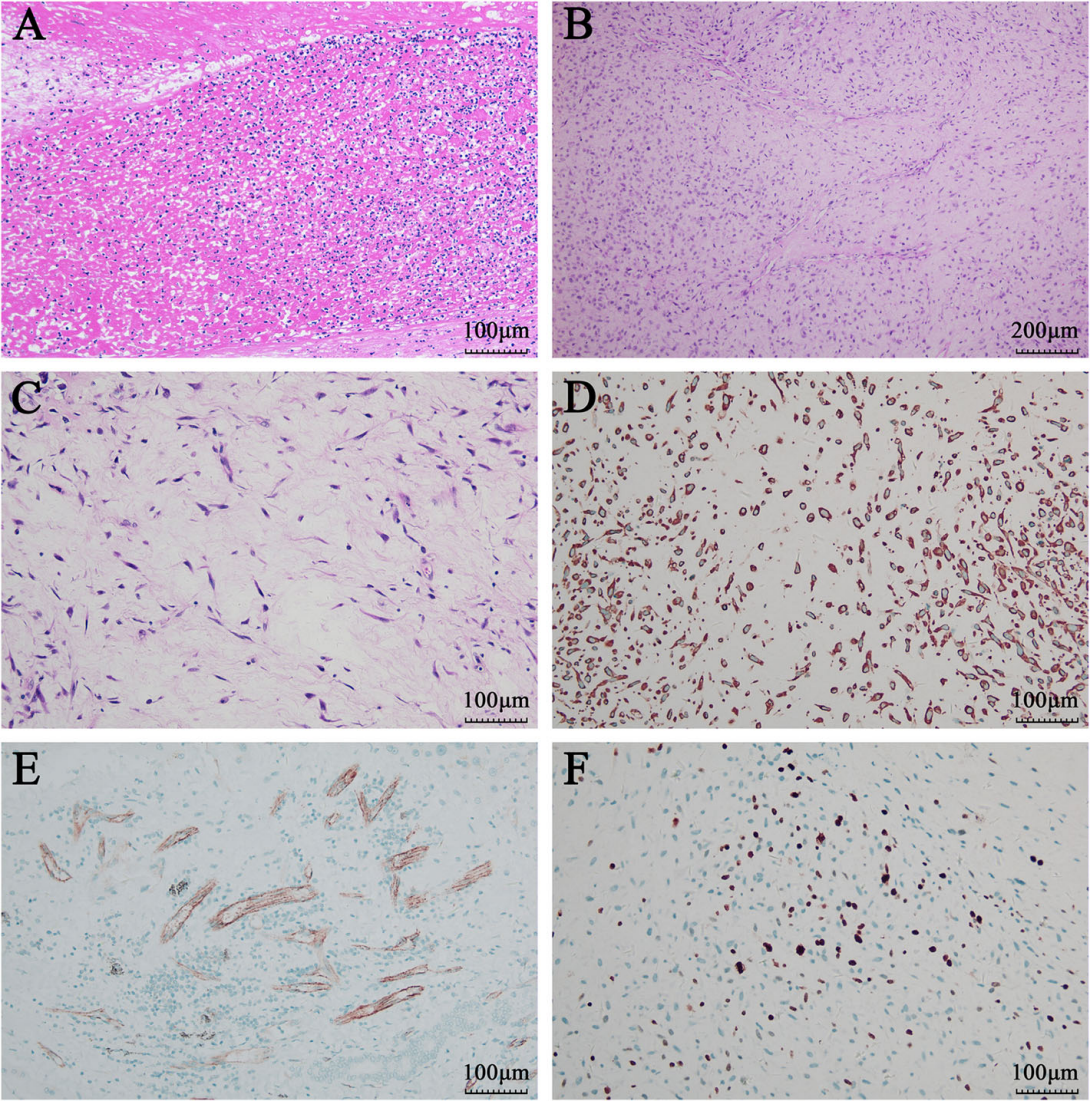
Hematoxylin-Eosin Staining and immunohistochemical staining after admission. Hematoxylin-Eosin Staining showing chronic inflammation of the gingiva lesion (**a**, ×200) and low-grade myxofibrosarcoma of the right mandible (**b**, ×100, **c**, ×200), immunohistochemical staining showing vimentin positive (**d**, ×200), CD31 positive (**e**, ×200), and Ki-67 positive (**f**, ×200)

Incisional biopsies of the gingival mass and the right mandibular lesion were performed under local anesthesia. Histopathology findings of the gingival lesion are shown in Fig. 4a. The gingival squamous epithelium was infiltrated with a large amount of plasma cells, neutrophils and other inflammatory cells. Abundant inflammatory exudation and fibrous tissue proliferation were also identified. Vascular cellulosic necrosis or granuloma was not obvious. PAS (Periodic Acid-Schiff stain) and methenamine silver stain showed no fungal organisms (figures not shown). These findings suggested chronic suppurative inflammation of the gingival mass. The cut surface of the mandibular lesion revealed an intraosseous nodular lesion. Histopathological exam of the mandibular biopsy showed scattered spindle and stellate cells with hyperchromatic nuclei distributed in a mucous matrix. Mildly atypical and mitotic figures were seen, while necrosis was not present (Fig. 4 b, c). Immunohistochemistry showed that the tumor cells were positive for vimentin (Fig. 4 d). Vascular CD31 stain showed the characteristic thin-walled and curvilinear vasculature (Fig. 4 e). The labeling index of Ki-67 was about 30% (Fig. 4 f). The tumor cells were negative for CK, S-100, P63, HHF-35 (figures not shown). Based on the clinical presentation, radiographic findings, histopathological and immunohistochemical examinations, the diagnosis of a low-grade myxofibrosarcoma of the mandible was made.

**Fig. 5:**
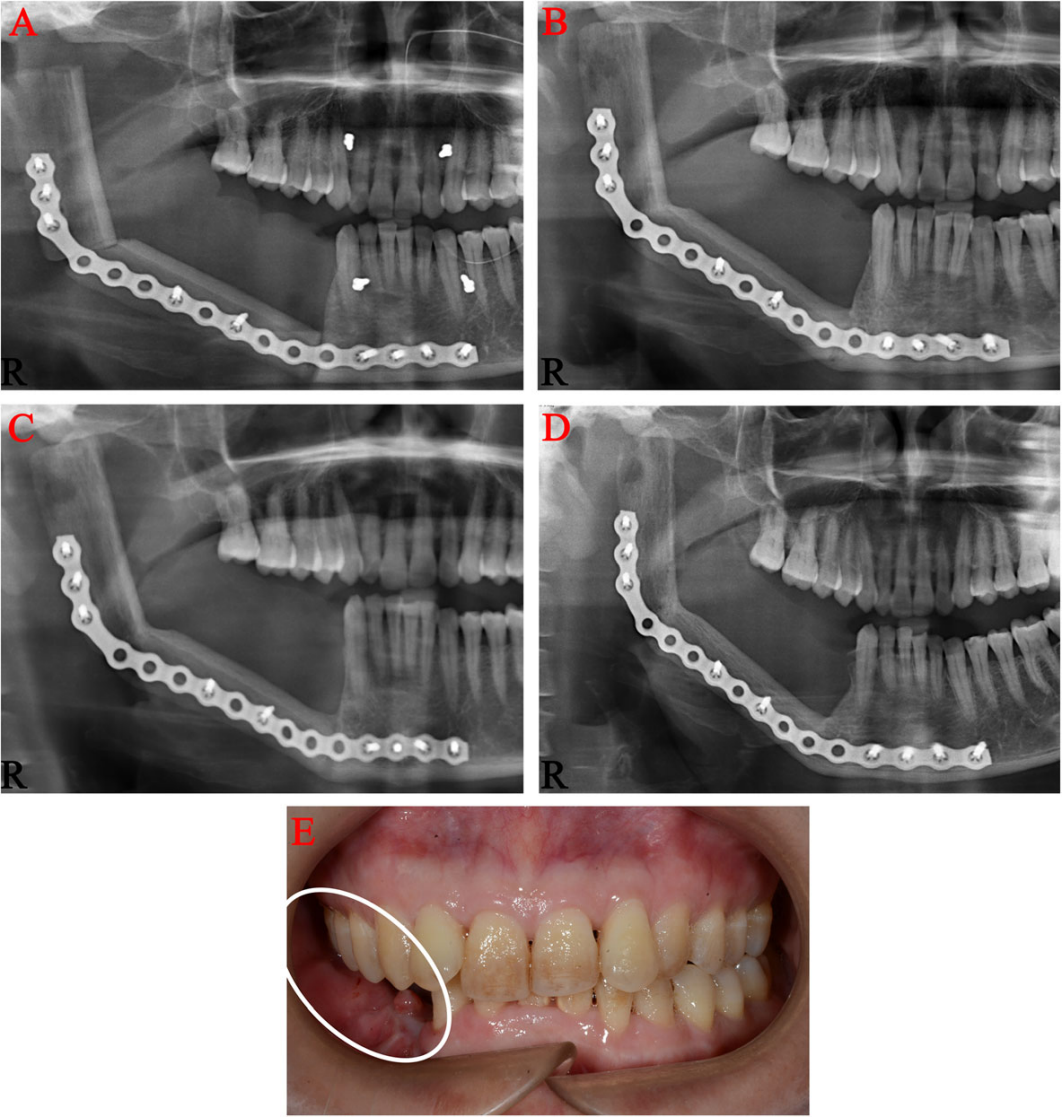
Postoperative panoramic radiographs and photographs of the patient. Panoramic radiographs of 1 week (**a**), 3 months (**b**), 6 months (**c**), and 20 months (**d**) after surgery showing no recurrence in the mandible; Postoperative photographs of 20 months (**e**) showing no recurrence in the surrounding soft tissues (the white box)

This patient was taken to the operating room for radical segmental resection of the right mandibular MFS with 2 cm margins, right supraomohyoid neck dissection, and immediate reconstruction with a left fibular ostocutaneous free flap. Due to the size of the lesion, a right mandibular hemimandibulectomy with disarticulation of the mandibular condyle was performed. The resected tissues were submitted for histopathologic and immunohistochemical examinations. None of the resected cervical lymph nodes were positive for malignancy. This patient was followed closely in the past twenty months and no local recurrence nor distant metastasis was detected to date (Fig. 5).

## Discussion and conclusions

The term myxofibrosarcoma (MFS) was first reported in the literature in 1951 [9]. It wasn’t until 1977, when Angervall et al. reported 30 cases of MFS that MFS became a clinically distinct diagnostic entity. According to the degree of cellularity, nuclear pleomorphism, and mitotic activity, MFS was categorized into four grades: I-IV [10]. In 1996, Mentzel et al. classified the tumor into low, intermediate, and high-grade MFS [11]. The 2002 third edition of the WHO Classification of Soft Tissue Tumors classified MFS as malignant fibroblastic/myofibroblastic tumors. Myxoid Malignant Fibrous Histiocytomas, for the first time, were considered a form of MFS [12]. The fourth edition WHO Classification of Soft Tissue Tumors regarding MFS was published in 2013 and the classification remained unchanged to date [13].

As a soft tissue sarcoma, MFS mainly occurs in the dermis or subcutaneous tissue, and occasionally occurs in the subfascial or intramuscular tissue [14]. The incidence of MFS in the head and neck region is quite rare [15]. The reported cases of MFS in the head and neck region were mostly in the soft tissues, such as the Schneiderian membrane of the maxillary sinus [16], lining of the sphenoid sinus [17], parotid gland [18], thyroid gland [19], and pharynx [20]. MFS occurring intra-osseously in the head and neck region is exceedingly rare. To the best of our knowledge, only two cases of intraosseous MFS were reported in the maxilla [1, 21] and four cases reported in the mandible [22–25] (Table 1). Most MFS tumor cells have the ultrastructural characteristics of a tumor of fibroblast differentiation and secretory activity. They commonly originate from fibrous connective tissue in the soft tissue, but the origin of MFS in bone tissue remains unclear. It is speculated that MFS may originate from the fibrous connective tissue in the endosteum or the mesenchymal tissue during tooth germ development.

**Table 1:** Cases of myxofibrosarcoma in the maxilla and mandible

Case report	Age (years) /Sex	Location	Chief complaint	Immunohistochemical features		Diagnosis	Treatment	Follow-up
Nakahara et al. [1]	52/M	Maxilla	Cheek discomfort and expansion of the upper right gums for 3 months	Pre-operation	myelin (±), synaptophysin (−), desmin (−), s-100 (−)	Inflammation reaction	Surgery, post-operative radiotherapy	Alive after 17 months
				Pre-operation	vimentin (+), Alcian blue (±), cytokeratin (−), s-100 (−)	MFS		
				Post-operation	vimentin (+), CK AE1/3 (−), CK 7 (−), CK 20 (−), S-100 (−), CK 34βE12 (−), desmin (−), a-SMA (−), ALK-1 (−)	MFS		
Quimby et al. [22]	72/F	Maxilla	A rapidly growing soft tissue mass in the posterior left maxilla	Pre-operation	NO	Peripheral giant cell granuloma	Pre-operative radiotherapy,surgery	Unknown
				Pre-operation	NO	Spindle cell sarcoma		
				Post-operation	NO	MFS		
Kummoona et al. [23]	35/M	Mandible	Numbness of the left lower lip of 2 months, mandilbe swelling	Pre-operation	NO	MFS	Surgery	Alive after 24 months
				Post-operation	NO	MFS		
Kargahi et al. [24]	61/M	Mandible	The fourth time of local recurrence	Pre-operation	NO	Unknown	Incomplete resection	Alive after 10 months
				Post-operation	vimentin (+), S100 (−), CK (−), Ki67 (1%)	MFS		
Zouloumis et al. [25]	23/M	Mandible	2-months history of a swelling on the left side of the lower face	Pre-operation	NO	Unknown	Surgery	Alive after 39 months
				Post-operation	NO	MFS		
Park et al. [26]	59/M	Mandible	Pain of posterior left mandible	Pre-operatio	NO	Myxofibroma	Surgery, chemo-radiotherapy	Alive after 12 months
				Post-operation	NO	MFS		

Like most soft tissue sarcomas, the pathogenesis of MFS is still unclear. It is suspected that multiple factors contribute to the development of MFS. Current research mainly focuses on the genetic characteristics of soft tissue sarcomas. It is found that MFS has a highly complex karyotype and shows gains and losses of numerous chromosomes or chromosome regions. Therefore, it is speculated that the pathogenesis of MFS may be related to chromosome abnormalities [26].

The clinical courses of MFS vary significantly and no specific clinical features can be identified. It often presents as a painless, slow-growing tumor. Low-grade MFS often demonstrates expansive growth, while high-grade MFS often shows local invasion or compression of the surrounding anatomical structures. High-grade MFS involving the airway or the major vasculature of the head and neck can lead to life-threatening complications.

The tumor in this case caused aggressive destruction of the right mandible, but without significant cortical expansion leading to facial asymmetry. The patient did not experience sensory alteration with the tumor involving the mandibular canal. The predominant symptom was the local swelling of the overlying gingiva. The referring dentist initially suspected inflammatory lesion of the oral mucosa, so radiographic examinations were not performed. The pathology report of the initial gingival biopsy suggested pyogenic granuloma. It wasn’t until the patient presented to our department (Department of Oral and Maxillofacial Surgery, Stomatological Hospital of Southern Medical University) for treatment, the necessary diagnostic radiographic examinations were performed. The radiographic findings prompted us to perform incisional biopsies of the gingival mass and mandibular lesion. The surgical pathology report supported the diagnosis of low-grade MFS of the mandible and chronic suppurative inflammation of the overlying gingiva.

MFS is associated with a relatively high rate of local recurrence or distant metastasis. Accurate preoperative diagnosis and proper treatment strategies are critical in managing patients with MFS. Preoperative imaging examinations including ultrasound, CT, and MRI can be used to determine the anatomical extent of the lesion involvement. Histopathologic examinations are considered the gold standard in establishing the diagnosis of MFS.

MFS is more likely to arise superficially in the subcutaneous tissue or dermis, than in deep soft tissues, such as the sub-fascial or intramuscular tissues. On gross examination, the superficial MFS tend to present as multinodular lesions along the epidermis or dermis and demonstrate variability in the gelatinous cut surfaces. The deep tissue MFS, on the other hand, often presents as a solitary mass with a grayish, fish-meat appearance with infiltrative borders.

Although the microscopic morphologies of MFS vary, there are some common histopathological features of MFS. These commonalities include varying degrees of myxoid (hyaluronic acid) matrixes composed of spindle to stellate cells that demonstrate nuclear atypia and pleomorphism, and curvilinear vasculature. According to the spectrum of cellularity, cytologic atypia, and mitotic activity, MFS is categorized into low, intermediate and high-grade.

In low-grade MFS, the tumor shows characteristics such as hypocellularity and prominent myxoid stroma on histology. Tumor cells are scattered and non-cohesive. They often present as plump spindle or stellate shaped cells with ill-defined and slightly eosinophilic cytoplasm and hyperchromatic nuclei arranged disorderly or in bundles. Mitotic figures are uncommon and tumor necrosis cannot be seen. In some cases, hyperchromatic multi-nuclear tumor cells can be seen. Occasionally, vacuolar and acidic mucous-filled pseudolipoblasts can also be seen, which are positive on AB-PAS stain. The essence of pseudoadipocyte is fibroblast, and there are no fat droplets in the cytoplasm. The characteristic thin-walled and curvilinear vasculature are prominent. In a few cases, the vasculature is clustered or branched. A variable number of inflammatory cells and tumor cells can be seen around the blood vessels [27, 28].

High-grade MFS is either partially or predominantly composed of solid sheets and cellular fascicles of spindle and pleomorphic tumor cells with marked nuclear and cytologic pleomorphism. Bizarre and multinucleated tumor giant cells with abundant eosinophilic cytoplasm and irregularly shaped nuclei are common. Mitotic figures, atypical cells, hemorrhage and coagulative necrosis are also common. Features such as prominent myxoid matrix and numerous curvilinear vessels can be found focally. Mucous degeneration and rich, slender capillary networks can be seen in the tumor stroma [27, 28].

The histopathological features of intermediate-grade MFS are between the low and high-grade MFS. Intermediate-grade MFS is more cellular and pleomorphic than the low-graded tumors. It lacks the extensive solid/sheet-like areas, pronounced cellular pleomorphism, and necrosis found in high-grade MFS. Mitoses are more commonly found than in low-grade tumors. Curvilinear vasculature is often prominent [27, 28].

In immunohistochemistry, the tumor cells of MFS are positive for vimentin. MSA and α-SMA can be focally positive, which indicate myofibroblast differentiation. CD31 is positive in tumor vasculature. S-100, desmin, caldesmon, keratin and histocyte markers such as CD68, Mac387, XIIIa are negative. Ki-67 is partially positive. Ki-67 has a positive correlation with tumor recurrence and can be used as an indicator of recurrence [27, 28].

Based on the clinical and radiographic findings described in this case report, the differential diagnoses include low-grade malignant fibromyxoid sarcoma, myxoid liposarcoma, myxoma, Nodular fasciitis, Spindle cell lipoma, and nerve sheath myxoma [27, 28]. Histopathological and immunohistochemical examinations are essential in establishing the correct diagnosis.

Although there are some variations in management of MFS, the primary treatment approach is radical surgical resection including a wide margin of adjacent disease-free tissue. Preoperative maxillofacial CT is necessary to determine the anatomical extent of the tumor and involvement of the surrounding tissue. It is generally believed that resection of at least 2 cm around the tumor is needed to ensure a negative surgical margin and reduce the chance of local recurrence and distant metastasis [29]. For the surgical site defect, pedicled or free tissue flaps can be used to reconstruct the forms and functions of the resected structures. Whether cervical lymph node dissection is necessary for head and neck MFS patients with clinically negative cervical lymph node remains unclear. In order to decrease the risk of local recurrence and distant metastasis, postoperative adjuvant treatment such as radiotherapy and/or chemotherapy can be added to the treatment regimen. In a randomized controlled trial (RCT) involving multiple subtypes of soft tissue sarcomas, it was found that radiotherapy helped to reduce the recurrence rate of the sarcomas [30, 31]. However, there are no RCTs specifically evaluating the effect of radiotherapy on MFS. Currently, there is no consensus on whether and when radiotherapy should be used in the management of MFS. In cases where complete resection was not possible or in patients with recurrent lesions, radiotherapy is recommended. Chemotherapy is commonly used in MFS patients with distant metastasis. Colia et al. found that chemotherapy agents such as the combination of anthracycline and isocyclophosphamide increase the survival rate in MFS patients. Other cohort studies did not support the same conclusion [32]. Considering the small sample sizes of the retrospective studies and the absence of RCTs, further studies on the risks and benefits of chemotherapy are still needed.

In this case report, this patient underwent radical hard and soft tissue resection with right hemimandibulectomy, disarticulation of the right mandibular condyle. The large mandibular defect was reconstructed with a fibular osteocutaneous free flap. Due to the consideration that MFS can metastasize to the adjacent cervical lymph nodes, supraomohyoid neck dissection was performed on the right side. The final surgical pathology report showed no cervical lymph node involvement, postoperative radiotherapy and chemotherapy were not prescribed.

Based on the current literature, the local recurrence rate for MFS does not correlate well with its histopathological grade. Most cases of MFS are low-grade lesions, and the short-term prognosis of this subtype is generally good. The local recurrence rate for a low-grade MFS is similar to that of a high-grade MFS. Low-grade MFS, however, can transform into a high-grade MFS after recurrence [33]. The local recurrence rate of MFS is about 16–57%. Multiple recurrences have also been reported and the rate for multiple recurrence is estimated between 25 and 52% [29]. Local recurrence has been reported as early as 2 months after resection and as far out as eight years postoperatively. The median recurrence time is about 27 months [34]. A negative surgical margin is the most important prognostic factor in estimating the local recurrence rate [34, 35]. The prognosis is generally poor for MFS that recurs within one year.

Distant metastasis is rarely seen in low-grade MFS. The rate of metastasis can be as high as 20 to 35% in intermediate and high-grade MFS. Distant metastases usually occur in the lungs and bones. The 5-year overall survival rate for MFS is between 61 and 77% [34]. Metastasis and mortality are closely associated with the histopathological grade of the MFS. For instance, metastasis is positively correlate with the depth of the tumor location, the size of necrosis areas, the size of tumor larger than 5 cm, and mucoid area less than 75% [21]. Patients with MFS need long-term follow-up to monitor for tumor recurrence. In this patient, if local recurrence and/or distant metastasis were detected in the future, reoperation, radiotherapy, chemotherapy or combined therapy will be considered.

In conclusion, besides odontogenic tumors, soft tissue malignancies such as MFS can occur in the mandible and cause the radiographic findings described in this case report. Recognizing the histopathological features of MFS and applying the appropriate immunohistochemical examinations are crucial in establishing the correct diagnosis. Wide Surgical excision with at least 2-cm margin of adjacent normal tissue and adjuvant postoperative radiochemotherapy are the primary treatment recommendations in managing patients with MFS. Long-term follow up for tumor surveillance is advisable in all cases. Many clinical questions such as the pathogenesis of the disease, tumor origin, efficacy of the adjuvant chemoradiotherapy remain unanswered. Further studies are necessary in improving our understanding of this malignancy which may help in providing better outcomes for our patients.

## Data Availability

Not applicable.

## Abbreviations

MFS: myxofibrosarcoma
WHO: The current World Health Organization
CT: computed tomography
RCT: randomized controlled trial
